# The General Internist

**Published:** 1994

**Authors:** Patrick G. O’Connor

**Affiliations:** Patrick G. O’Connor, M.D., M.P.H., is an associate professor of medicine at Yale University School of Medicine, New Haven, Connecticut

## Abstract

Although general internists recognize the medical complications arising from alcohol abuse, they may be less familiar with its social and psychological consequences or with methods for its treatment. Because internists are the only physicians some patients see, they must be prepared to diagnose and take part in the treatment of alcohol problems.

Alcoholism^1^ has a significant impact on the practice of general internal medicine (Barnes et al. 1987; National Institute on Alcohol Abuse and Alcoholism [NIAAA] 1990; Institute of Medicine [IOM] 1990; Fleming and Barry 1992). In the advanced disease, which is most widely recognized by internists, alcoholism’s symptoms include such medical complications as cirrhosis, hepatitis, or hypertension. Perhaps more challenging, however, is identifying the common problem of early stage alcohol abuse, which may not cause any symptoms and thus is missed on routine examination (IOM 1990). General internists should be equipped to recognize alcoholism in all of its forms, from asymptomatic through advanced disease, and be able to participate in managing patients along the entire spectrum of alcoholism (Barnes et al. 1987; NIAAA 1990; IOM 1990; Fleming and Barry 1992).

This article reviews some of the important epidemiologic and clinical features of alcoholism and offers practical advice on its diagnosis and management for internists.

## Epidemiology of Alcoholism

Although community-based surveys have found that the prevalence of alcoholism in the general U.S. population can be as high as 12 percent, its prevalence is higher in populations of patients seen in medical settings (Barker and Whitfield 1991). For example, studies have shown that approximately 20 to 30 percent of patients seen in primary care settings may have problems with alcohol, including alcoholism (Rydon et al. 1992; Barry and Fleming 1993). When data from hospital services are examined, alcoholism has been documented in as high as 42 percent of male and 35 percent of female patients (Lewis and Gordon 1983). Given that these prevalence figures are similar to those for other chronic diseases, such as hypertension and diabetes, internists should evaluate their patients for alcoholism with the same rigor that they approach these other common problems.

## Evaluating Patients for Alcoholism

Despite the high prevalence of alcoholism, internists often fail to identify it in patients, perhaps because of inadequate training in detecting the disorder, which often is asymptomatic. They also may miss alcoholism in some patients because of the prevailing negative attitudes about substance abuse, which suggest that substance abuse is not part of the realm of the general internist and that treatment is ineffective (Barker and Whitfield 1991; Rydon et al. 1992; Hays and Spickard 1987). Thus, general internists must overcome considerable barriers if they are to recognize alcoholism in their patients. Internists can identify alcoholism, however, when they consider incorporating the following suggestions into their practices (table 1).

### Patient History

When evaluating patients, the patient history is the most valuable source of information. The internist should include the following two questions as part of the history: (1) Do you ever drink alcohol? and (2) Do you have a family history of alcoholism? The first question provides an initial assessment of alcohol use and indicates whether a more detailed screening is necessary. An answer of “No” in the presence of alcohol-related medical or social problems suggests the possibility of denial and a need to revisit this issue with the patient. The second question helps determine if the patient is at increased risk for alcoholism (Barker and Whitfield 1991; Hays and Spickard 1987), because people with alcoholic relatives stand a greater chance of developing alcohol problems themselves.

The internist should ask patients who do drink more detailed questions (table 2) about their drinking patterns and screen for alcoholism if necessary (discussed below). Routine alcohol-use questions help determine patients’ drinking patterns, including frequency, amount, and whether they consider themselves to have had drinking problems in the past. The internist should keep in mind that alcohol use has its own social context that may vary significantly among people, depending on factors such as nationality and ethnic background. Information on the frequency of drinking may help distinguish daily drinkers from binge drinkers,^2^ both of whom may develop patterns associated with alcoholism. When determining alcohol intake, the internist should explore a range by asking patients about their usual number of drinks and whether they ever drink more, to identify binge drinking.

### Physical Examination

Unlike a patient history, a physical examination is not an effective method for detecting unsuspected alcoholism; there are no diagnostic physical signs of alcoholism, and most patients who have a history of problem drinking will have a normal physical examination. However, the examination is a useful tool. The presence of certain common medical conditions, such as hypertension, gastrointestinal bleeding, and chronic liver disease, which are associated with alcoholism, can raise a “red flag” in the mind of the internist to the possibility of alcohol abuse (Barnes et al. 1987). In addition, in patients with suspected or known alcoholism, the physical examination can reveal other alcohol-related complications (discussed below).

### Laboratory Tests

No laboratory test effectively detects alcoholism in asymptomatic patients. In one study of hospitalized patients that compared the efficacy of the CAGE questionnaire, a well-validated screening instrument, with laboratory markers (see the article by Salaspuro, pp. 131–135), the CAGE test had a positive predictive value^3^ of 62 percent as compared with a positive predictive value of approximately 30 percent for laboratory markers (Bush et al. 1987).

Certain laboratory abnormalities, such as elevated liver enzymes and macrocytic anemia—a condition characterized by a reduced number of red blood cells that also are enlarged—can suggest alcoholism (Barnes et al. 1987). For example, a test that measures levels of the liver enzyme gamma-glutamyl transferase (GGT) is the most sensitive of the liver function tests. It indicates current alcohol intake, but test results can be affected by conditions other than alcohol use. Laboratory tests also can be helpful in evaluating patients with known or suspected alcoholism for alcohol-related complications.

Based on the results of the physical examination, laboratory tests, and the patient’s answers to the history questions related to alcohol use, the internist can determine whether the patient should be screened for alcoholism.

### Screening Tools

Instruments such as questionnaires (e.g., the CAGE test) that screen for alcohol use disorders are among the more effective tools internists may rely on to help determine whether a patient has developed alcoholism (Buchsbaum et al. 1991; Cyr and Wartman 1988). Several instruments for screening alcohol use disorders have been developed and evaluated. (For a description of common screening tools and terminology, see Nilssen and Cone, pp. 136–139.)

It is important to remember that screening instruments for alcoholism—as with other screening tools such as mammography for breast cancer—do not establish a diagnosis of alcoholism. A positive result on the CAGE test does indicate that further evaluation is needed to determine if alcoholism is present, just as a biopsy may be indicated in the case of an abnormal mammogram. Thus, patients with known or suspected alcoholism should be asked more specific questions.

### Use of Other Substances

Because patients suspected of having alcohol problems also may be abusing other substances, the internist must consider polysubstance use when evaluating patients who use alcohol. These patients should be asked about their use of tobacco, prescription drugs, and illicit drugs. Recognizing polysubstance use without asking about it may be difficult because its physical signs, if any, are similar to those of alcohol use. For example, some characteristics of alcohol addiction, including tolerance, withdrawal, loss of control, and social consequences, are common to other substance addictions. However, alcohol is distinct from these other substances given the specific pharmacological properties that result in a pattern of withdrawal and characteristic medical complications.

### Making the Diagnosis

Having ascertained the patient’s level of alcohol use employing the techniques above, the general internist can focus on making a diagnosis of alcoholism. The internist should consider the characteristic features of alcoholism, including the patient’s inability to control alcohol intake, preoccupation with alcohol, use of alcohol despite the presence of adverse consequences, and distortions in thinking such as denial (Morse and Flavin 1992). The internist also should refer to the definitions and diagnostic criteria of alcohol abuse and alcohol dependence outlined in the *Diagnostic and Statistical Manual of Mental Disorders, Fourth Edition* (DSM–IV; American Psychiatric Association [APA] 1994). Diagnostic criteria for alcohol abuse include (1) a pattern of pathologic alcohol use (e.g., a need for daily use to function, an inability to cut down, and binges) and (2) impairment in social functioning due to alcohol use (e.g., employment, legal, and family problems). Diagnostic criteria for alcohol dependence include indications of alcohol abuse and of tolerance, as evidenced by increasing amounts of alcohol intake or withdrawal (APA 1994).

Even with these specific criteria, the internist often must consider nonspecific symptoms (e.g., fatigue or anxiety) in the presence of a positive family history or other problems commonly thought to be alcohol related (e.g., hepatitis or pancreatitis) when diagnosing alcoholism.

## Common Complications of Alcoholism

Although patients who abuse alcohol often are asymptomatic, alcohol abuse is known to result in a variety of social, behavioral, medical, and psychiatric complications that can serve as clues to the presence of problem drinking (Morse and Flavin 1992; APA 1994; Eckardt et al. 1981).

### Social and Behavioral Complications

Often, internists are more aware of the most frequently described medical and psychiatric complications of alcohol abuse, such as chronic liver disease and depression, than they are of the social and behavioral complications. These problems, however, may be the most common presenting “symptoms” of alcohol abuse seen by the internist. Such symptoms often are revealed during a routine examination and can be quite diverse, including indications of family dysfunction, legal and employment problems, and frequent accidents (see the article by Soderstrom, pp. 127–130).

### Medical Complications

Although many patients with alcohol problems do not have medical complications, some do exhibit nonspecific symptoms such as fatigue, abdominal pain, and poor nutrition, which may be caused by any of a number of alcohol-related complications (table 3).

#### Gastrointestinal Tract

Problems of the esophagus, such as chronic inflammation, malignancies, Mallory-Weiss tears,^4^ and esophageal varices,^5^ all have been associated with alcohol abuse (Eckardt et al. 1981; Van Thiel et al. 1981). Presenting symptoms can include weight loss; chest pain, or “heartburn”; vomiting of blood; and difficulty in swallowing.

Alcohol abuse has been associated with gastrointestinal bleeding from peptic ulcer disease (Eckardt et al. 1981) along with nausea, vomiting, and abdominal pain.

Alcohol abuse also can lead to malnutrition due either to poor eating habits or to malabsorption of nutrients. These nutritional deficiencies can be evident in a patient with weight loss, peripheral neuropathy (due to a folate deficiency), and Wernicke’s encephalopathy (due to a thiamine deficiency).

#### Liver

Alcohol abuse is associated with “fatty liver,” which may be asymptomatic or associated with such nonspecific symptoms as abdominal discomfort or anorexia. The period of time spent drinking required for developing fatty liver is highly variable among people.^6^

Alcoholic hepatitis represents more advanced acute liver disease as evidenced by fever, nausea, vomiting, abdominal pain, and liver dysfunction (Lieber 1984). Although chronic liver dysfunction occasionally is asymptomatic, patients with more advanced dysfunction, such as cirrhosis, may have jaundice, weight loss, and bleeding.

#### Pancreas

Among the most dramatic manifestations of alcohol abuse is acute pancreatitis, which causes intense abdominal pain, nausea, vomiting, and fever (Eckardt et al. 1981; Van Thiel et al. 1981). Patients with recurrent pancreatitis may develop chronic pancreatitis, manifested by chronic intractable abdominal pain and poor nutritional status from malabsorption of nutrients.

#### Nervous System

Alcohol can have acute and chronic toxic effects both on the central nervous system (the brain and spinal cord) and the peripheral nervous system (outside the brain and spinal cord) (Charness et al. 1989). Although acute central nervous system effects such as intoxication and withdrawal are seen commonly in emergency settings, internists also may see these effects when managing patients in primary care settings. Alcohol abuse may be associated with central and peripheral nervous system effects such as mild to severe cognitive impairment, including impaired short- and long-term memory and deficient functioning in activities of daily living, such as an uncoordinated gait, which is evidence of cerebellar degeneration (Charness et al. 1989).

#### Cardiovascular System

Common cardiovascular manifestations of alcohol abuse include hypertension with fatigue, palpitations, and shortness of breath; cardiac arrhythmias (or irregularities in the heartbeat, e.g., “holiday heart”); and chronic cardiomyopathy (or disease of the heart muscle) (Lang and Quinnunen 1987). Research data suggest that moderate to high levels of chronic alcohol intake are associated with hypertension and that decreased alcohol intake may lower blood pressure (Klatsky et al. 1977; Puddey et al. 1987). Despite evidence of the harmful cardiovascular effects of alcohol, other data suggest that moderate alcohol intake may have beneficial effects, such as reducing fats found in the blood serum and thus potentially reducing cardiac risk (Gaziano et al. 1993). Any benefit, however, may well be outweighed by the risks of the other alcohol-related complications previously described.

#### Other Medical Complications

Cancers known to be alcohol related include those of the mouth, oropharynx, and esophagus (Lieber et al. 1979). Cancers postulated to be associated with alcohol abuse include those of the pancreas, colon, and breast, although data for these cancers have been less convincing. Alcohol-related cirrhosis also has been associated with the development of primary liver cancer, or hepatoma.

Other important medical problems seen in alcohol-abusing patients result from a weakened immune system and include the infectious diseases pneumonia and tuberculosis (Adams and Jordan 1984). These diseases may be influenced by alcohol-related factors such as poor nutrition, respiratory tract dysfunction, and the environment within which alcoholics may live.

### Psychiatric Complications

The general internist may see alcoholic patients who have a variety of psychological symptoms or psychiatric diagnoses. Symptoms that can be strongly associated with alcoholism include fatigue, anorexia, or mild depression; however, they often indicate conditions unrelated to alcohol problems.

It is suggested that between 30 and 50 percent of alcoholics may meet criteria for major depression (Goodwin 1992). Patients may receive a diagnosis of alcoholism in addition to a diagnosis of a specific psychiatric disorder. For example, anxiety disorders are common in alcoholics (Goodwin 1992). It is important, therefore, that internists perform a careful psychiatric evaluation on these patients, and when appropriate, refer patients for more specialized care.

## Discussing the Diagnosis With Patients

### Handling Barriers to Discussion

Among the most challenging aspects of the practice of internal medicine is discussing a diagnosis of alcoholism with a patient (Barnes et al. 1987; Barker and Whitfield 1991; Hays and Spickard 1987). Patient attitudes toward or perceptions of alcoholism may create barriers to the discussion, which must be overcome if the patient is to get treatment. One barrier is the feeling of shame and hopelessness common in patients with alcoholism. Because of these feelings, it is imperative that the internist discuss the diagnosis in a sensitive and nonjudgmental fashion and be hopeful with patients, assuring them that their problem can be treated successfully.

As a step toward opening a discussion, the internist should educate patients about the effects of alcohol and the diagnosis and prognosis of alcoholism. Patients may have their own preconceived notions about the “skid row” alcoholic, another barrier to discussion, and may not understand that there is a spectrum of disease severity. Patients may feel more comfortable discussing alcoholism as a “chronic disease” and need to learn that it is not indicative of a personality disorder or moral inadequacy.

Once patients are educated about alcoholism, they must learn to recognize that they have the disease. Physicians can use information gathered from the history, physical examination, laboratory studies, and screening tests to demonstrate that the patients’ clinical problems correspond with those indicative of alcoholism. To make this point to patients, physicians may tell them how their particular social or occupational dysfunction or medical complications relate to patterns of pathological drinking (Barnes et al. 1987). Patients must be left with a sense that the physician will be their advocate in helping them deal with the psychological and medical consequences of alcoholism.

Particularly challenging may be the patients who do not accept the diagnosis, despite exhibiting major complications of alcoholism. In these situations, physicians must continue to work with patients to overcome their denial so that treatment may be attempted. Future visits to treat other medical problems may serve as opportunities for the physician to readdress alcoholism by further educating patients and discussing the diagnosis.

It is important for the physician to differentiate between patients who deny that they have a problem from those who are ambivalent about the diagnosis, because the means for getting these patients into treatment differ. Ambivalent patients may recognize that their own situation is indicative of alcoholism but may not believe that the diagnosis represents a significant problem. In this case, motivation to seek treatment is as important as education about the diagnosis. Patients who develop concerns about their drinking may wish to restrict their treatment activities to those that can be provided by their internist because of their anxiety about the potential stigmatization associated with entering a formal treatment program. However, the physician must let the patient know from the beginning that more specialized treatment may be necessary eventually.

### Readiness for Change

An important aspect of successful treatment is a patient’s readiness for change, which can be assessed by the physician. According to a model for change developed by Prochaska and DiClemente (1986), patients may be thought of as attaining various levels of willingness to change an alcohol problem. The levels range from precontemplation—when the patient has not yet thought about the need to change, to maintenance—when the patient has made a change and continues to manage the problem by sustaining a healthier lifestyle. Once physicians have determined which level a patient has reached, they may find techniques that rely on open-ended questions and emphatic listening helpful in moving a patient from one level to another (Miller and Rollnick 1991). The overall goal in this process is to avoid a relapse and secure long-term maintenance of abstinence from alcohol use.

## Treating Alcoholism

Through activities such as using brief office-based interventions, referring patients to treatment programs, involving the patients’ families in treatment, and monitoring patients’ progress (discussed below), internists may play a critical role in their patients’ alcoholism treatment (table 1).

### Office-Based Interventions

A growing body of evidence suggests that office-based interventions can have a significant impact on decreasing alcohol use in patients with alcohol problems (Barnes et al. 1987; IOM 1990; Barker and Whitfield 1991; Babor et al. 1986). These brief minimal interventions typically include advice or counseling given in the provider’s office. For example, after reviewing the patient’s drinking pattern and evidence of drinking-related complications, the physician may advise the patient to stop drinking or reduce alcohol intake. Brief interventions may be useful for all patients with problem drinking, although many patients, especially those with moderate or severe disease, also may require referral to alcoholism treatment.

### Involving Family in Treatment

Whether a treatment strategy is office based or a patient is referred to an outside agency, it may be helpful to involve patients’ families in the treatment process when possible. This is particularly important given that a supportive environment is often the key to successful treatment. With the patient’s consent, family members should be invited to a discussion with the physician about the diagnosis and treatment plans for the patient. Physicians should be aware of the potential for conflict in this situation; however, the potential benefits may outweigh this risk. Formal family therapy available through an alcoholism treatment center may be particularly helpful to patients whose families are supportive and want to be involved in the treatment process. Family members also may benefit from participating in a support group such as Al-Anon.

### Referring Patients for Alcoholism Treatment

In addition to office-based interventions, patients with moderate to severe alcoholism often will require referral to outside services. When a referral is made, it is critical for the internist to communicate closely with the alcoholism treatment professionals so that comprehensive and coordinated services can be provided to patients. Self-help groups such as Alcoholics Anonymous (AA) can be attractive to some patients, given their accessibility and low cost (Barnes et al. 1987). In any city, there is often more than one meeting every day, and no fee is charged.

The majority of patients with alcohol-related problems can be managed in outpatient settings such as AA. Internists should be familiar with available outpatient treatment programs that provide services such as individual and group counseling to help patients maintain abstinence.

However, patients who have failed multiple attempts at outpatient treatment may benefit from a trial of inpatient therapy. This approach may be indicated for people with a high level of alcohol dependence or significant co-occurring medical or psychiatric disorders that would make outpatient detoxification unmanageable.

### Monitoring Recovery of Patients in Treatment

Once patients have entered treatment successfully, the internist can continue to play an important role in monitoring their recovery (Barnes et al. 1987; NIAAA 1990; Barker and Whitfield 1991). The internist can be instrumental in explicitly supporting abstinence (Barnes et al. 1987; Barker and Whitfield 1991) by regularly inquiring whether the patient has attained abstinence and providing appropriate positive feedback. The internist also should continue to communicate with the patient’s alcoholism treatment professionals to remain abreast of the patient’s progress.

The internist’s role also may include managing the patient’s alcohol-related medical problems over a number of months or years and looking for evidence of impending relapse (Barnes et al. 1987; Barker and Whitfield 1991). Accordingly, internists should be aware of the progression a patient’s attitudes often follow. Immediately after giving up alcohol, patients may experience a significant degree of euphoria as a reaction to their newly attained abstinence. Relapse becomes more likely for patients in whom distressing psychological symptoms—such as anxiety, for which they may have been drinking alcohol originally—become exacerbated in the absence of alcohol. One indication of increased risk of relapse is a protracted withdrawal syndrome, which can last months and cause depression, anxiety, and insomnia (NIAAA 1990). Reassurance from the internist is particularly important for these patients, whereas psychoactive medications (e.g., benzodiazepines) to treat chronic withdrawal symptoms should be avoided because of the alcoholic’s susceptibility to becoming addicted to such medications.

Even patients who initially avoid relapse find other pitfalls awaiting them as they try to remain abstinent. For example, they may experience difficulty reestablishing the appropriate relationships necessary to support abstinence. Recovery may require them to find jobs or change jobs and develop new friendships with nonalcoholic people. The internist can reinforce the necessity of these changes.

## Summary

The diverse medical and psychological problems associated with alcoholism have a significant impact on the practice of internal medicine. Internists must acknowledge the possible existence of alcohol as the cause of problems among their patients and should screen all patients for evidence of drinking problems. Accordingly, they must be proficient in using specific screening and diagnostic strategies. Initial alcoholism treatment may take place in primary care settings by internists who should foster a noncritical environment and be prepared to help patients recognize their alcoholism and begin to address it. Internists should be well versed in local treatment resources and know how to refer patients when needed. Ultimately, a primary care physician such as an internist may be best suited for coordinating the care of alcoholic patients and should keep in close contact with both patients and treatment professionals throughout the period of alcoholism treatment.

## Figures and Tables

**Table 1:** Role of the General Internist in the Evaluation and Treatment of Alcoholism

Evaluation of the Patient
Take a patient history.
Perform a physical examination—look for conditions that signal physical damage as a result of alcohol abuse.
Obtain laboratory tests.
Administer screening tests.
Arrive at a diagnosis.

Discussion of the Diagnosis
Overcome the patient’s barriers to acceptance of the diagnosis.
Educate the patient about alcohol’s effects and alcoholism’s prognosis.
Help the patient recognize that he or she has the disease.
Determine the patient’s level of motivation/readiness to change.

Office-Based Brief Interventions
Offer advice and counseling to the patient.
Involve the patient’s family in treatment.

Referral to Treatment Programs
Select the appropriate type of program—outpatient, residential, partial hospitalization, or hospital based.
Communicate with treatment specialists to ensure comprehensive and coordinated patient care.

Oversight of Recovery
Support the treatment effort.
Monitor the patient for evidence of impending relapse.

**Table 2:** Questions About Alcohol Use That Physicians Can Ask Their Patients When Taking a History

Ask all patients: Do you ever drink alcohol? Do you have a family history of alcoholism?
Ask all patients who drink alcohol: Alcohol use questions When was your last drink? How often do you drink? How much do you usually drink? Do you ever drink more? Have you ever had a drinking problem? Screening questions from the CAGE test Have you ever felt you should Cut down on your drinking? Have people Annoyed you by criticizing your drinking? Have you ever felt bad or Guilty about your drinking? Have you ever had a drink first thing in the morning to steady your nerves or to get rid of a hangover (Eye opener)?
Ask patients who do or may have problem-drinking behaviors about more specific aspects of their alcohol use: Withdrawal symptoms and complications Tolerance Medical complications gastrointestinal disease cardiovascular disease neurological disease other complications Social complications family problems such as divorce and physical abuse employment problems such as loss of job legal problems related to such events as traffic crashes Loss of control
Ask patients who drink alcohol about their use of other substances: Tobacco Prescription drugs Illicit drugs such as heroin and cocaine

**Table 3:** Medical Complications of Alcoholism and Their Clinical Signs and Symptoms

Complications	Signs and Symptoms
Gastrointestinal Tract	
Esophageal disease	Difficulty in swallowing
	Chest pain (“heartburn”)
	Vomiting of blood
	Weight loss
Stomach/Bowel	Nausea
	Vomiting
	Abdominal pain
	Diarrhea
	Weight loss
	Peripheral neuropathy^1^ (from folate deficiency)

Liver	
Fatty liver	Asymptomatic
	Mild abdominal discomfort
	Anorexia
Alcoholic hepatitis	Fever
	Nausea
	Vomiting
	Abdominal pain
	Liver dysfunction
	Jaundice
Cirrhosis	Weight loss
	Bleeding abnormalities
	Edema (swelling)

Pancreas	
Acute pancreatitis	Intense abdominal pain
	Nausea
	Vomiting
	Fever
Chronic pancreatitis	Intractable abdominal pain
	Weight loss
	Diarrhea

Central Nervous System	Intoxication
	Withdrawal
	Wernicke’s encephalopathy^2^
	Seizures
	Dementia
	Cerebellar dysfunction (e.g., loss of muscular coordination)

Peripheral Nervous System	Numbness
	Weakness

Cardiovascular System	Fatigue
	Hypertension
	Palpitations
	Shortness of breath
	Arrhythmia (irregular heartbeat)

1 Peripheral neuropathy is a set of functional disturbances or pathological changes in the peripheral nervous system that cause symptoms such as numbness and weakness.

2 Wernicke’s encephalopathy is a neurological disorder characterized by confusion, apathy, drowsiness, and lack of muscular coordination when walking. The disorder is caused by a deficiency of the vitamin thiamine and most commonly results from chronic alcohol abuse.

## Footnotes

1 Although the terms “alcoholism” and “alcohol abuse” usually are not interchangeable, they are used here and throughout the article as general terms meaning chronic overconsumption of alcohol.

2 In this context, a binge drinker is an episodic heavy drinker who consumes several drinks on any single occasion.

3 Positive predictive value is the probability that a person who tests positive on a screening test for alcoholism actually is an alcoholic.

4 Mallory-Weiss tears are tears in the opening from the esophagus to the stomach.

5 Esophageal varices are an enlargement of the branches of a vein connected to the lower esophagus. These branches are prone to breaking open, and patients bleed severely when this occurs.

6 Editor’s Note: Whereas the period of time required for the development of fatty liver is variable, some findings have described relatively brief periods of heavy drinking causing fatty liver (Rubin, E., and Lieber, C.S. Alcohol-induced hepatic injury in nonalcoholic volunteers. *New England Journal of Medicine* 278(16):869–876, 1968).
